# Conserved HA-peptide NG34 formulated in pCMV-CTLA4-Ig reduces viral shedding in pigs after a heterosubtypic influenza virus SwH3N2 challenge

**DOI:** 10.1371/journal.pone.0212431

**Published:** 2019-03-01

**Authors:** Marta Sisteré-Oró, Júlia Vergara-Alert, Thomas Stratmann, Sergi López-Serrano, Sonia Pina-Pedrero, Lorena Córdoba, Mónica Pérez-Maillo, Patrícia Pleguezuelos, Enric Vidal, Veljko Veljkovic, Joaquim Segalés, Jens Nielsen, Anders Fomsgaard, Ayub Darji

**Affiliations:** 1 IRTA, Centre de Recerca en Sanitat Animal (CReSA, IRTA-UAB), Campus de la Universitat Autònoma de Barcelona, Bellaterra, Barcelona, Spain; 2 Department of Cell Biology, Physiology and Immunology, Faculty of Biology, University of Barcelona, Barcelona, Spain; 3 Centre for Multidisciplinary Research, Institute of Nuclear Sciences VINCA, Belgrade, Serbia; 4 UAB, Centre de Recerca en Sanitat Animal (CReSA, IRTA-UAB), Campus de la Universitat Autònoma de Barcelona, Bellaterra, Barcelona, Spain; 5 Departament de Sanitat i Anatomia Animals, Facultat de Veterinària, UAB, Bellaterra (Cerdanyola del Vallès), Barcelona, Spain; 6 Virus Research and Development Laboratory, Department of Virus and Microbiological Special Diagnostics, Statens Serum Institut, Copenhagen S, Denmark; University of South Dakota, UNITED STATES

## Abstract

Swine influenza viruses (SIVs), the causal agents of swine influenza, are not only important to control due to the economic losses in the swine industry, but also can be pandemic pathogens. Vaccination is one of the most relevant strategies to control and prevent influenza infection. Current human vaccines against influenza induce strain-specific immunity and annual update is required due to the virus antigenic shift phenomena. Previously, our group has reported the use of conserved hemagglutinin peptides (HA-peptides) derived from H1-influenza virus as a potential multivalent vaccine candidate. Immunization of swine with these HA-peptides elicited antibodies that recognized and neutralized heterologous influenza viruses *in vitro* and demonstrated strong hemagglutination-inhibiting activity. In the present work, we cloned one HA-peptide (named NG34) into a plasmid fused with cytotoxic T lymphocyte-associated antigen (CTLA4) which is a molecule that modifies T cell activation and with an adjuvant activity interfering with the adaptive immune response. The resulting plasmid, named pCMV-CTLA4-Ig-NG34, was administered twice to animals employing a needle-free delivery approach. Two studies were carried out to test the efficacy of pCMV-CTLA4-Ig-NG34 as a potential swine influenza vaccine, one in seronegative and another in seropositive pigs against SIV. The second one was aimed to evaluate whether pCMV-CTLA4-Ig-NG34 vaccination would overcome maternally derived antibodies (MDA). After immunization, all animals were intranasally challenged with an H3N2 influenza strain. A complete elimination or significant reduction in the viral shedding was observed within the first week after the challenge in the vaccinated animals from both studies. In addition, no challenged heterologous virus load was detected in the airways of vaccinated pigs. Overall, it is suggested that the pCMV-CTLA4-Ig-NG34 vaccine formulation could potentially be used as a multivalent vaccine against influenza viruses.

## Introduction

Influenza-like disease in pigs started occurring in both United States and Europe in connection with the human influenza pandemic in 1918. Proof of this is the close relationship between the early H1N1 swine viruses with the human influenza virus of 1918, as determined by genetic analyses [1].

The influenza disease in pigs, although rarely fatal, has a high morbidity close to 100% [2]. The etiological agents of the disease, Swine influenza viruses (SIVs), are considered one of the major causes of acute respiratory disease outbreaks in swine herds [3,4]. Together with the well-known postulate that pigs act like “mixing vessels” of new reassortant influenza viruses [5], it may be of vital importance to develop effective vaccines that can confer protection against a broad umbrella of different SIV subtypes.

SIVs co-circulating in European swine are from the subtypes H1N1 (SwH1N1), H3N2 (SwH3N2) and H1N2 (SwH1N2) [6,7] that are also equivalent to the more prevalent subtypes in North America [8]. Thus, in line with the circulating subtypes in the swine population [6–8], current commercial vaccines available for pigs consist of two or three inactivated SIV strains belonging to the aforementioned subtypes. Vaccines are commonly administered in pregnant sows to stimulate passive antibody transfer via colostrum. Regrettably, protection with these commercial vaccines is only achieved when the strain either closely or completely matches with the challenged virus [9–13]. The reported lack of protection against divergent strains is thought to be associated with the poor stimulation of the mucosal and cellular immunity provided by inactivated-typed vaccines [11,14,15]. It is thus fundamental to seek new vaccine strategies eliciting robust immune response and protection against drifted or emerging strains occurring from antigenic drift or antiviral shift, respectively.

The new emergent vaccine formulas must take maternally derived antibodies (MDA) interference into account since the presence of MDA inhibit/neutralize current vaccines [16,17]. Even though optimal vaccination of animals should begin at the time of disappearance of maternal antibodies, this approach is often unfeasible due to a high degree of variability in antibody titers between individuals [18]. Similarly, inactivated-typed vaccines may pose drawbacks in piglets with MDA by suppression of the antibody responses [19–21], and because they may enhance respiratory diseases upon influenza infection [22,23] or cause an aggravated pneumonia called vaccine associated enhanced respiratory disease (VAERD) [24]. Thus, an additional aim of this work was to evaluate the capacity of the vaccine to provide protection against a heterologous influenza virus strain to individuals with MDA.

Our group has previously reported the use of an HA1-based peptide (NF-34) in pigs as potential vaccine [25]. Partial virus clearance after an intranasal challenge with the homologous pH1N1 (pandemic swine-origin A/Catalonia/63/2009 H1N1 IV) influenza virus was observed along with a strong humoral and T-cell response in animals vaccinated with the NF-34 HA-peptide. Although NF-34 HA showed concomitant detection of antibody response it did not totally correlate with neutralizing activity [25]. In the present study, NF-34 peptide has been modified (named NG34) and employed in a DNA vaccine approach (pCMV-CTLA4-Ig-NG34). In addition, to ameliorate safety and large-scale vaccination approaches, a needle-free intradermal administration technique (IDAL device) was applied.

The research objectives were two-fold in two different swine experiments depending on the initial presence/absence of MDA. Firstly, to assess vaccine efficacy against a heterologous SIV isolate in seronegative animals (SwH3N2) and, secondly, to investigate whether farm animals could clear a heterologous virus challenge, thus overcoming MDA obstacles.

In the present work, we demonstrated that immunization of seronegative pigs with pCMV-CTLA4-Ig-NG34 antigen formulation induced neutralizing antibodies that inhibited hemagglutination of a heterologous SIV. Moreover, immunized conventional farm pigs (both the influenza virus seropositive and seronegative) were fully or partially protected against a heterologous influenza virus challenge as they either completely eliminated or significantly reduced virus secretion and cleared the virus from the respiratory airways.

## Materials and methods

### Ethics statement

All animal studies presented in this work were approved by IRTA’s Ethics Committee for Animal Experimentation and the Animal Experimentation Commission from the Catalonia Government (Spain) in compliance with the Directive, EU 63/2010, the Spanish Legislation (RD 53/2013) and the Catalan Law 5/1995 and Decree 214/1997. Treatment with anesthetics or analgesics was not considered because animals did not suffer from the disease and/or experimental manipulation.

### Experimental design

**Fig 1:**
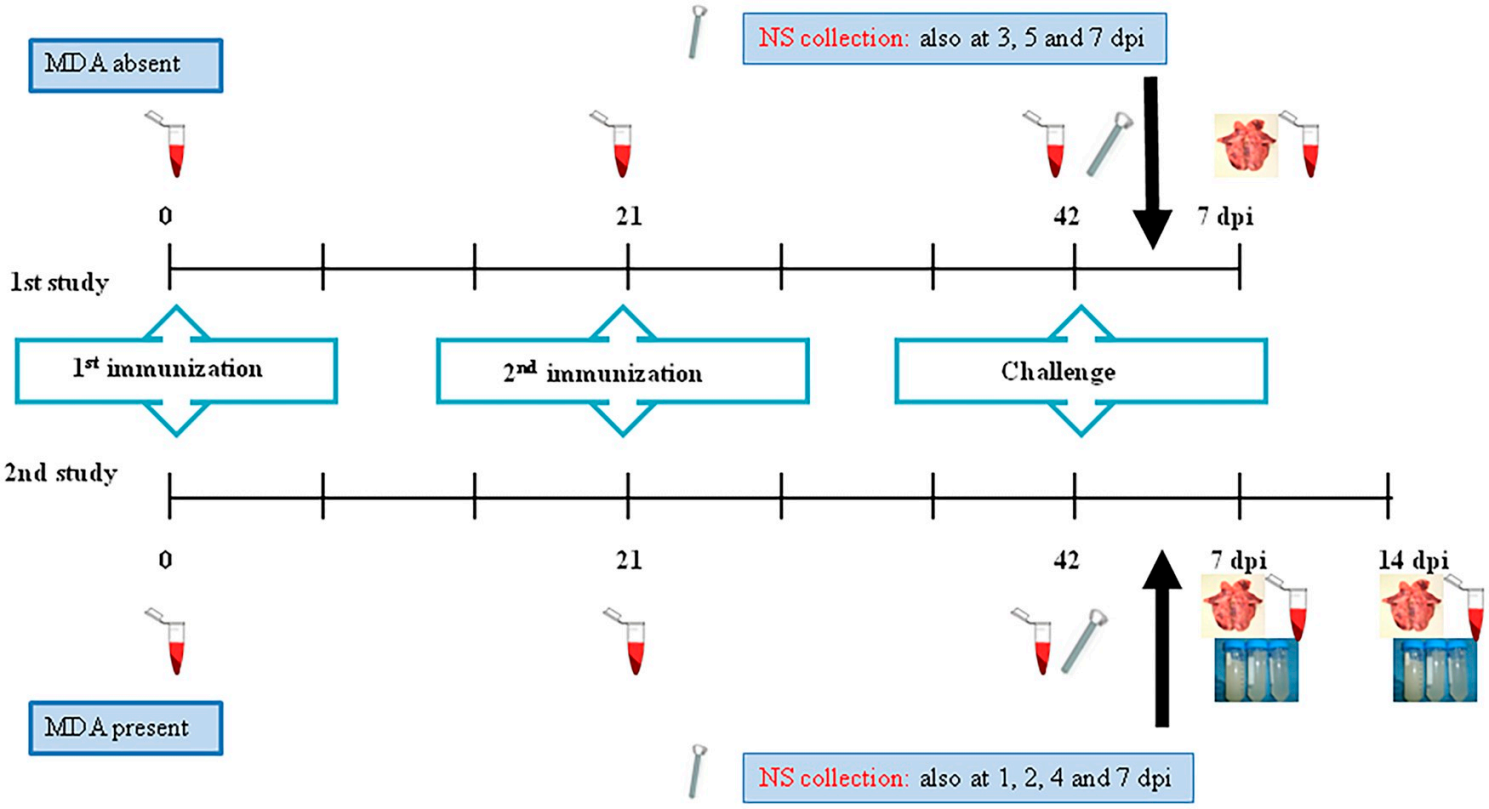
Experimental outline of two studies. (A) 1^st^ study outline: seronegative pigs against SIV were vaccinated at 0 and 21 days, and challenged at 42 days. Sera were collected each pre-vaccination, pre-challenge and at necropsy day (7 dpi), as indicated. Lung tissues at 7 dpi. Nasal swabs collection took place at challenge day and at all days indicated in the figure. (B) 2^nd^ study outline: seropositive pigs against SIV, vaccinated at 0 and 21 days, and challenged at 42 days. Sera were collected each pre-vaccination, pre-challenge and necropsy days (7 and 14 dpi). Lung tissues were obtained at 7 and 14 dpi. In this case, bronchoalveolar fluids (BALFs) were also collected at 7 and 14 dpi. Nasal swabs collection took place at challenge day and at all days indicated in the figure. Dpi, days post-inoculation; NS, nasal swabs.

The results presented in this manuscript are representative of two almost identical experimental studies performed in swine, whose outlines are described in Fig 1. In both studies, animals were observed daily during the course of the experiments by monitoring for flu-like clinical signs and rectal temperature profiles; and the severity of clinical signs was assessed from 0 to 3 according to a previously described scoring [26]. Animals received water and food *ad libitum*.

In a first study, 10 five-to-six week-old, Influenza A virus seronegative, Yorkshire x Landrace pigs were used. Animals were housed, vaccinated and challenged in the animal Biosafety Level 3 (BSL-3) facility at CReSA (Barcelona, Spain). Animals were randomly distributed into two groups of 5 animals each (Group A = non-vaccinated pigs, animals 1–5; Group B = vaccinated pigs, animals 6–10). After an acclimatization period of 5 days, five pigs were vaccinated twice with an interval of 3 weeks by injecting the final plasmid formulation using a needle-free device, Intra Dermal Application of Liquids, (IDAL MSD Animal Health), on the dorsal side of the back of each animal as previously described [27]. The final plasmid formulation used for immunization consisted of 600 μg DNA construct pCMV-CTLA4-Ig-NG34 (applied in 3 shots/vaccination) mixed with Diluvac Forte from MSD Animal Health (1:1 v/v). The remaining five pigs were sham-vaccinated (control group). Two weeks after the second vaccination, both groups received an intranasal challenge of a SIV H3N2 isolate at a concentration of 10^6^ TCID_50_/mL in 3 mL saline solution into each animal (1.5 mL/nostril) by using the MAD device (Intranasal Mucosal Atomizing Device, Teleflex). Euthanasia and necropsies were carried out 7 days post-inoculation (dpi). Sera from all individuals were collected previous to each immunization, before the challenge, and at 7 dpi. Nasal swabs were collected before challenge and at 3, 5 and 7 dpi. Eventually, lung tissues were obtained and fixed by immersion in 10% neutral buffer formalin to perform the histopathological analysis.

The second study was performed applying identical conditions as those of the first study, albeit with influenza A virus seropositive animals. The goal of this second study was to simulate conditions generally occurring under usual conventional farm conditions. For that reason, animals were vaccinated in the farm and were transported to BSL-3 facilities for challenge. Each group consisted of six animals (Group A = non-vaccinated pigs, animals 1–6; Group B = vaccinated group, animals 7–14), and three pigs were euthanized at either 7 or 14 dpi. Sample collection was similar in both studies until day 7, albeit at different times. In this study, nasal swabs were collected before challenge and at 1, 2, 4 and 7 dpi. Moreover, bronchoalveolar fluids (BALFs) were collected from the right lung of each pig at the time of euthanasia (7 and 14 dpi).

A complementary study with three SIV-seronegative pigs was performed in order to evaluate the immunogenic effect of the plasmid without the NG34 sequence (empty vector). The same experimental design as the described for the study 1 was carried out (Fig 1), but animals were vaccinated with 600μg DNA construct pCMV-CTLA4-Ig. Here, BALFs were also collected at 7 dpi.

### Cells, virus and antigens

Madin-Darby Canine Kidney (MDCK, ATCC CCL-34) cells were cultured in Dulbecc’s Modified Eagle Medium (DMEM), supplemented with 10% fetal bovine serum (FBS), 1% penicillin/streptomycin and 1% L-glutamine. The cell cultures were kept at 37°C with 5% CO_2_ atmosphere in a humidified incubator.

SwH3N2 (A/swine/Spain/003/2010 H3N2 IV) [GenBank JQ319724 and JQ319726] influenza virus was used in this study for the intranasal challenge. The 50% tissue culture infectious dose (TCID_50_) was calculated in MDCK cells according to Reed and Muench method [28].

The sequence identity between antigen of interest (NG34) and challenge virus, SwH3N2, used in this study is of 26% (Table 1).

**Table 1:** Amino acid sequence of NG34 (peptide of interest) and the HA from the challenged virus (A/swine/Spain/001/2010 (H3N2).

Antigen	Aminoacid sequence
**NG34**	NSDNGT**CYP**G**D**FID**Y**EE**LR**EQLS**S**VSSF**E**RFEIF
**HA from A/swine/Spain/001/2010 (H3N2)**	KA -FSN**CYP**Y**D**VPE**Y**TS**LR**SLIA**S**SGTL**E**FTNED

The amino acid identity between sequences is depicted in bold.

Purified hemaggluinins of A/California/04/09(H1N1)pdm09 and A/Aichi/2/1968 (H3) were purchased from SinoBiological (Sino Biological, cat. no. 40340-V08B and 11707-V08H; respectively) and were used as antigens.

### Sample collection

Nasal swabs collected at predetermined time points were placed in 500 μL of PBS with antibiotics (100 U/mL penicillin and 0.1 mg/mL streptomycin). Serum samples were obtained from the jugular vein. After necropsy, BALFs from animals from the second study were also obtained by introducing 150 mL of PBS into the right lung of each pig, massaged gently and recollected into 50 mL falcon tubes [29]. BALFs were further centrifuged to remove cells. All supernatant samples were stored at -80°C until analysis.

### Pathological assessment

Complete necropsies were performed, with special emphasis on macroscopic examination of lung parenchyma. Moreover, samples from apical, medial and cranial part of diaphragmatic pulmonary lobes were taken and fixed by immersion in 10% buffered formalin. Lung tissues were then embedded in paraffin, cut in 5 μm sections, stained in hematoxylin and eosin, and the severity degree of broncho-interstitial pneumonia was scored in a blinded fashion by a single pathologist using established criteria [30].

### Plasmid synthesis

Two amino acids were replaced from the original HA1-based peptide (NF-34) to design the NG34 peptide (Table 2). NG34 peptide derives from the HA1 protein of the pH1N1 A/Catalonia/63/2009 strain [GenBank: ACS36215] and was theoretically predicted by the Informational Spectrum Method (ISM), and mapped within the flanking region of the HA1. In order to enhance immunogenicity, the NG34 peptide sequence was reverse-translated and cloned into an expression vector encoding human IgG fused with the extracellular domain of CTLA4. EndoFree plasmid gigakit (Qiagen, Barcelona, Spain) was used for purification and large-scale plasmid production. The plasmid was resuspended in sterile saline solution and stored at -20°C until use.

**Table 2:** Amino acid modifications in peptide NF34 to obtain the NG34 peptide.

Peptide	Aminoacid sequence
NF34	NS**E**NGTCYPGDFIDYEELREQLSSVSSFE**K**FEIF
NG34	NS**D**NGTCYPGDFIDYEELREQLSSVSSFE**R**FEIF

The amino acids differences between sequences are depicted in bold type.

### Quantitative real time RT-PCR (RT-qPCR)

A TaqMan RT-qPCR was carried out to determine and quantify viral RNA in nasal swabs and BALFs collected at different time-points during the study. Extraction of RNA was carried out using NucleoSpin RNA isolation kit (Macherey-Nagel), according to the manufacturer’s instructions. Primers and probes used in this study, one-step RT-PCR Master Mix Reagents (Applied Biosystems) and amplification conditions ran in a Fast7500 equipment (Applied Biosystems) to amplify the conserved fragment of the matrix (M) gene of Influenza viruses are described elsewhere [31]. Samples in which fluorescence was undetectable were considered negative.

### Subtypic quantitative real time RT-PCR (RT-qPCR)

Viral RNA extracted with NucleoSpin RNA isolation kit (Macherey-Nagel) was tested to amplify a 95 bp fragment of the hemagglutinin (HA) gene of the challenged strain A/swine/Spain/003/2010 H3N2 IV. Based on a previous work, specific primers for the subtype H3 were designed [32]: forward 5’-TCCTTTGCCATATCATGCTTTTTG-3, and reverse 5’-ATGCAAATGTTGCACCTAATGTTG-3’. Specificity of primers was checked utilizing BlastN [33] through the Influenza Research Database (https://www.fludb.org/brc/blast.spg?method=ShowCleanInputPage&decorator=influenza) [34]. Real time amplification was performed employing the Power SYBR Green RNA-to-CT 1-Step Kit (Applied Biosystems, P/N #4389986) following manufacturer’s indications and using 2 μL of eluted RNA in a total volume of 20 μL. In brief, for RNA-to-C_T_ 1-Step, the real-time PCR was performed using a Fast7500 equipment (Applied Biosystems, Foster City, CA, USA) and following cycles: 48°C for 30 min (for cDNA synthesis), 95°C for 10 min (transcriptase inactivation), followed by 95°C for 15 s and 60°C for 1 min for 40 cycles. Dissociation curve (melting curve) analyses were performed employing the parameters of a hot start at 60°C for 15 s and measuring the fluorescence every 0.5°C until 95°C to confirm specific amplification.

Prior to the setting up of the RT-qPCR, standards were constructed. The amplification conditions for 95 bp of the HA fragment were: a reverse transcription at 50°C for 30 min; an initial denaturation reaction at 95°C for 15 min and 40 PCR-cycles of 94°C 30 s, 55°C 1 min and 72°C 1 min. The obtained HA gene fragment amplicon was cloned into pGEMT vector (Promega Madison, WI, USA) and transformed by heat shock in *Escherichia coli* TOP10 competent cells (Invitrogen, Paisley, UK). The recombinant plasmid was purified using the QIAprep Spin kit (Qiagen) and quantified by using BioDrop *μLITE* Spectrophotometer (BioDrop Ltd, Cambridge, UK). The copy number of recombinant plasmid was calculated as described elsewhere [35] by following the formula: N (molecules per μL) = (C (DNA) μg/μL/K (fragment size in bp)) × 182.5 × 10^23^ (factor derived from the molecular mass per the Avogadro constant). Serial 10-fold dilutions of known concentration were made and the standard curves were generated using copies of the recombinant plasmid harbouring the HA gene fragment from the SwH3N2 isolate.

### Immunoassays

Virus-antigen specific serum antibodies were detected by ELISA. The influenza virus proteins used to detect specific antibodies were hemagglutinins (HA) from A/California/04/09(H1N1)pdm09 and A/Aichi/2/1968(H3N2). Briefly, ELISA plates (Costar, Corning Incorporated) were coated overnight with 2 μg/mL recombinant influenza hemagglutinin protein antigen in sodium bicarbonate (50 mM) buffer at 4°C. Blocking was performed using 3%BSA/PBS for 1 hour at room temperature following washes with 1% Triton X-100/PBS. Sera were diluted 1:100 in the blocking buffer and added to the 96 well plates during an incubation period of 1 hour at room temperature. Then, plates were washed four times and incubated during 30 minutes at 37°C with an anti-pig IgG (whole molecule)-Peroxidase (Sigma) diluted 1:10000 with the washing buffer. Plates were again washed four times with 1%Triton X-100 PBS and 50 μL of TMB was added during 8 to 10 minutes for the enzymatic reaction. Finally, the reaction was stopped by adding 50 μL of 1 N H_2_SO_4_. All samples were analyzed in triplicates.

### Hemagglutination inhibition assay (HAI)

Hemagglutination inhibition assay was performed following the standard procedures described previously [25]. Positive and negative sera against H3N2 were purchased from GD Animal Health (Holland). All sera were analyzed in duplicate.

### Statistical analysis

Mean and standard deviations were calculated with Excel 2007 (Microsoft Office) and statistical differences between the groups were calculated. Briefly, all data obtained were first normalized by Shapiro-Wilk test and later the groups were compared using either the t-test (in case of normally distributed data), or the Wilcoxon test (in case of non-normally distributed data). All calculations were carried out using R statistical software (http://cran.r-project.org/).

## Results

### Clinical and pathological evaluation

In the first study, one animal from unvaccinated group (animal 5) manifested fever at 4 and 7 dpi. From the vaccinated group, fever could be detected in four out of five pigs; three of them (animals 6, 7 and 8) had fever during days 2, 3 and 4 post-inoculation and one of them (animal 9) on days 3 and 4. No other clinical signs could be observed, except one vaccinated animal (animal 8), which was coughing at 4 and 5 dpi. Likewise, four out of six animals had fever in the unvaccinated group (animals 1, 2, 5 and 6) and three out of six from the vaccinated group (animals 7, 8 and 11) from the second study. Also, one animal from each group was coughing (animal 1 and 14, both at 3 dpi), and only one vaccinated pig (animal 7) showed apathy and a loss of weight after the challenge.

**Fig 2:**
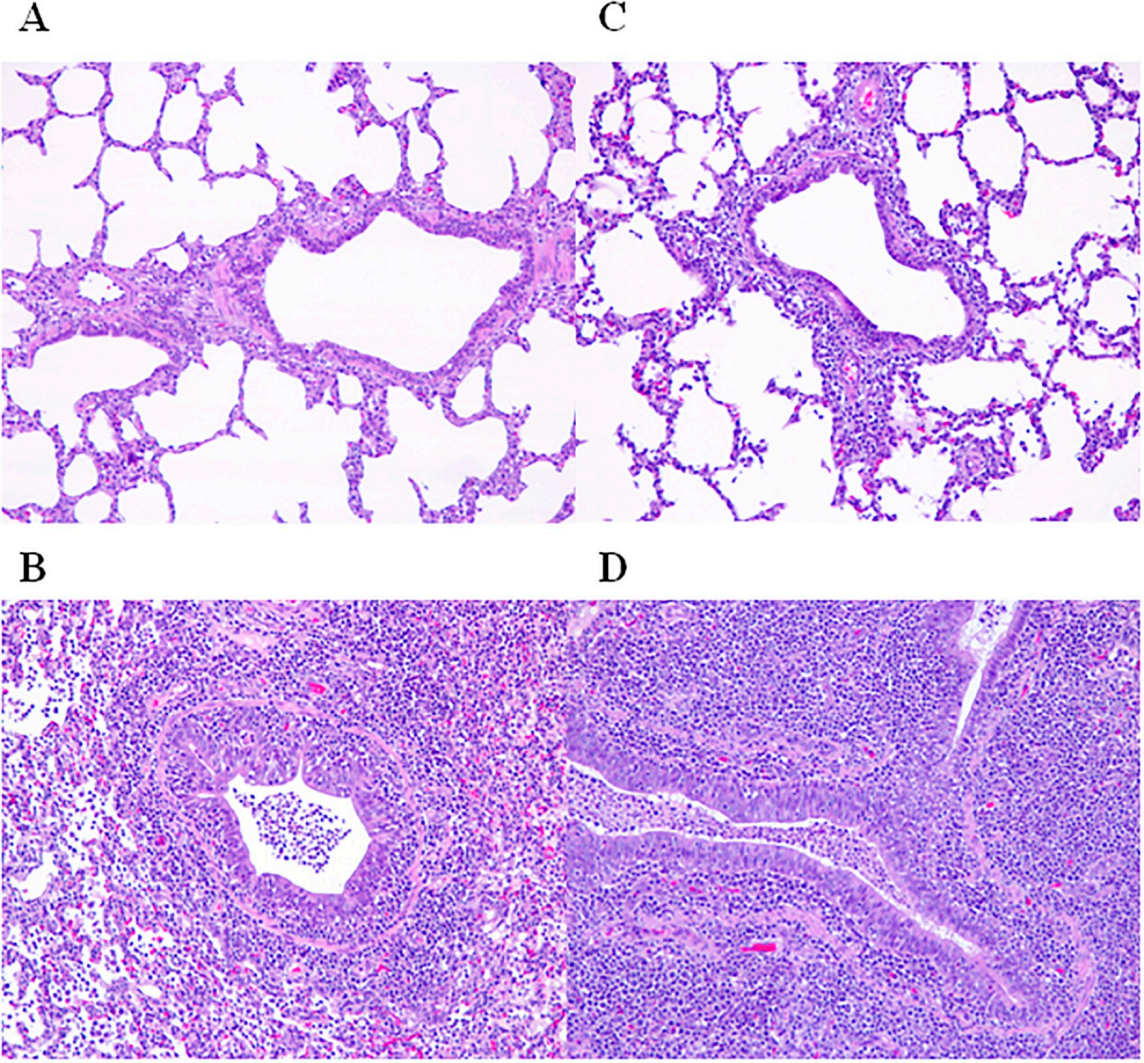
Representative sections from lung samples fixed with formalin and embedded in paraffin, stained with hematoxylin and eosin and microscopically evaluated following the scoring system [30] (magnification 100x): (A) scoring 0, (B) scoring 1, (C) scoring 2, and (D) scoring of 3.

In the first study, no differences in the histological lesions in the lung tissues were found at 7 dpi between groups.. No differences in the severity of the histological lesions in the lung tissues were detected at 7 or 14 dpi in the second study (Table 3). Fig 2 shows different sections of histological lung tissues illustrating the different scoring values (0, 1, 2 or 3). Additionally, besides the broncho-interstitial pneumonia scoring, other pathological findings were also recorded. In the first study, animal 8 had bronchiolitis fibrosa obliterans. In the second study, suppurative bronchopneumonia was present in one animal from the unvaccinated group (animal 1) and in 3 out of 6 animals from the vaccinated group (animals 7, 9 and 10); pig 8 had fibrous pleuritis.

**Table 3:** Scoring and observations given to each hematoxylin-eosin stained preparation for every single pig of 2^nd^ study.

Vaccine applied	Animal identification	Necropsy date	Broncho-interstitial pneumonia
**Unvaccinated**	1	7 dpi	2
	2	7 dpi	2
	5	7 dpi	3
	3	14 dpi	0.5
	4	14 dpi	1.5
	6	14 dpi	0.5
**Vaccinated**	7	7 dpi	3
	9	7 dpi	3
	12	7 dpi	2
	8	14 dpi	2
	10	14 dpi	0.5
	11	14 dpi	1

### Immunization with pCMV-CTLA4-Ig-NG34 eliminates or significantly reduces viral shedding

**Fig 3:**
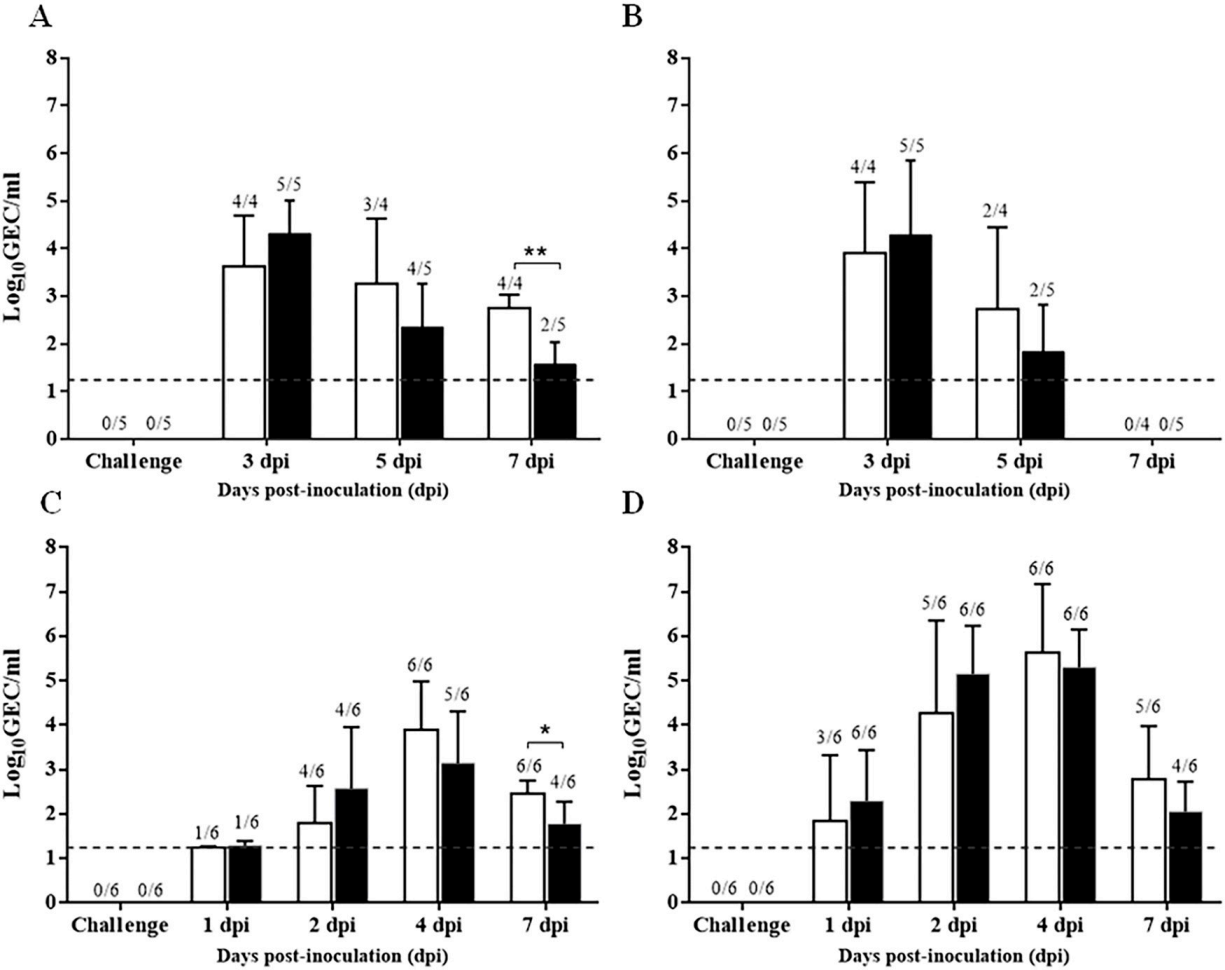
Viral detection in nasal swabs samples by RT-qPCR. Mean values of **genomic equivalent copies (GEC) per mL** obtained from nasal swabs samples (1^st^ study) collected at 0, 3, 5 and 7 dpi, from seronegative animals and (B) subtypic RT-qPCR results from the 1^st^ study (C) Mean values of GEC per mL obtained from nasal swabs samples (2^nd^ study) collected at 0, 1, 2, 4 and 7 dpi. from seropositive animals and (D) subtypic RT-qPCR from the 2^nd^ study. White bars correspond to Group A (unvaccinated group) and black bars to Group B (pCMV-CTLA4-Ig-NG34 vaccinated group). Dpi, days post-inoculation; Dashed lines indicate the detection limit of the assays: 1.24 log_10_GEC/mL. Error bars indicate the mean ± SEM.

In comparison to the control group, pigs immunized twice with pCMV-CTLA4-Ig-NG34 showed reduced viral shedding within the first week after challenge in both studies (Fig 3). The mean of genomic equivalent copies (GEC) per mL of the vaccinated pigs was inferior to the mean of the unvaccinated group at 5 and 7 dpi (Fig 3A). Remarkably, in the first study (seronegative pigs) from the vaccinated group, in three out of five animals no viral RNA was detected at 7 dpi. By contrast, viral RNA could be detected in all the five pigs from the unvaccinated group (*p*<0.01) (Fig 3A). Moreover, one animal from the unvaccinated group (animal 3) died, most likely due to a secondary bacterial infection. A reduction in the subtypic RNA shedding was also observed at 5 dpi in pCMV-CTLA4-Ig-NG34 vaccinated seronegative animals; however, no virus was detectable in the vaccinated and non-vaccinated animals on day 7 post-inoculation. (Fig 3B).

In animals with MDA (Study II; seropositive pigs), the mean of GEC per mL was lower at 4 and 7 dpi than those observed in the unvaccinated group. Noteworthy, at 7 dpi, in two out of the six animals (animals 8 and 9) we could not detect viral RNA (*p*<0.05) (Fig 3C). Regarding subtypic viral RNA in MDA positive animals, at 4 and 7 dpi, the mean of GEC per mL, was lower than those of the unvaccinated group. Furthermore, at 7 dpi viral RNA could not be detected in two vaccinated animals (Fig 3D).

Results from the vaccinated pigs with pCMV-CTLA4-Ig (empty plasmid) showed that none of the three pigs were reducing the viral shedding at 7 dpi (S1 Table).

### Humoral response to a heterologous influenza virus after pCMV-CTLA4-Ig-NG34 vaccination

To determine whether the NG34 peptide of H1N1 origin could confer protection against a heterosubtypic circulating influenza strain, pigs were challenged with the H3N2 influenza virus subtype. In addition, attempting to potentially improve the vaccine efficiency, we decided to deliver the antigen in a DNA vaccination approach instead of peptide. Immunogenicity of the vaccine was examined by the presence of specific antibodies, raised against H1 and H3, and their ability to HAI against the challenged heterologous virus in the sera collected from vaccinated and non-vaccinated animals. Specific immune response in the BALFs collected from MDA seropositive animals (from the 2^nd^ study) was also examined by testing for specific H1 and H3 antibodies.

**Fig 4:**
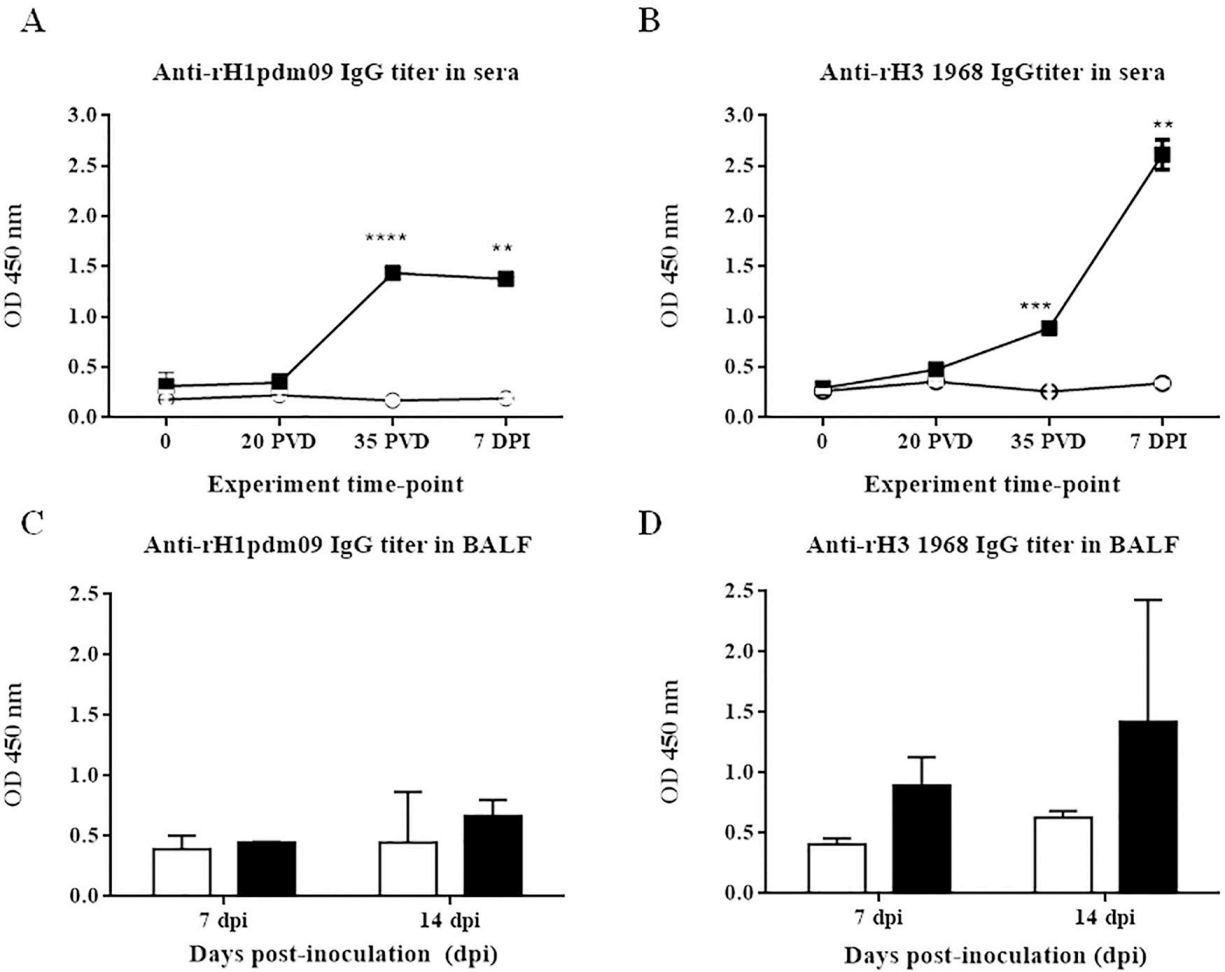
Serum antibody HA-specific IgG titers detected in sera and BALFs samples by ELISA test. Mean of serum antibody levels detected in all individuals at time-points 0, 20 PVD and 35 PVD, and 7 dpi of Groups A and B (A) against HA from A/California/04/09(H1N1)pdm09, and (B) against HA from A/Aichi/2/1968(H3N2) are shown. Mean of BALFs antibody levels detected in pigs necropsied at 7 and 14 dpi of Groups A and B (C) against HA from A/California/04/09(H1N1)pdm09, and (D) against HA from A/Aichi/2/1968(H3N2). White circles/bars designate group A (unvaccinated group), and black squares/bars designate group B (pCMV-CTLA4-Ig-NG34 vaccinated group). OD, optical density. PVD, post-vaccination days and Dpi, days post-inoculation. Error bars indicate the mean ± SEM. Statistically significant differences between vaccine treatment groups (P value <0.05) are marked with **: *p*<0.01, ***: *p*<0.001, ****:*p*<0.0001.

pCMV-CTLA4-Ig-NG34 immunization in pigs elicited antibodies that were recognizing both the HA1 and HA3 hemagglutinin subtypes (Fig 4A and 4B). Increased antibody levels were observed at 35 post-vaccination days (PVD, pre-challenge) in the vaccinated group (Fig 4A and 4B). The levels of antibodies were higher against HA1 (Fig 4A), since NG34 belongs to HA1 subtype. Furthermore, sera from all vaccinated animals, collected 7 days after the intranasal challenge with SwH3N2 influenza virus, manifested a potent boost in H3 subtype-specific antibodies in comparison to non-vaccinated SwH3N2 influenza virus infected control group (Fig 4B).

Additionally, we evaluated the HA1 and HA3 subtypes HA-specific IgG titers in BALFs samples. While not statistically significant, vaccinated animals achieved a greater antibody titer at the two necropsy points of the 2^nd^ study, with increased values at 14 dpi (Fig 4C and 4D). The difference encountered was greater for antibodies against the HA3 subtype among groups and, in a higher rate, likely due to the fact that an H3 virus was used for the challenge.

None of the pigs vaccinated with pCMV-CTLA4-Ig (empty plasmid) raised antibodies against H3 subtype before or after the challenge against SwH3N2 (S1 Table).

**Fig 5:**
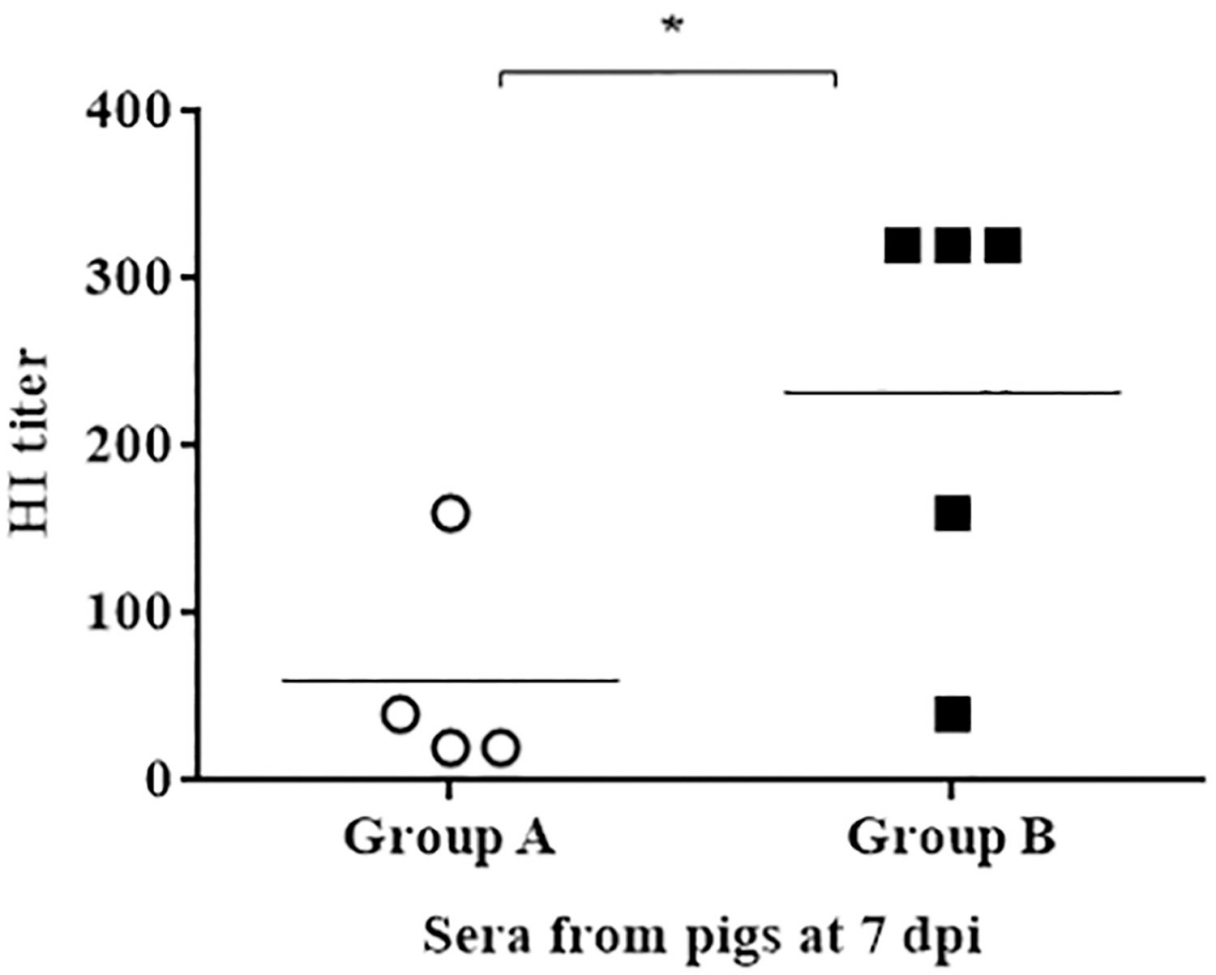
HAI activity at 7 dpi against SwH3N2 in sera from seronegative pigs (Study 1). HI titer obtained with sera from unvaccinated (Group A) and vaccinated (Group B) pigs, at 7 dpi against the SwH3N2. White circles designate group A (unvaccinated group), and black squares designate group B (pCMV-CTLA4-Ig-NG34 vaccinated group). HI: Inhibition of the hemagglutination. Error bars indicate the mean ± SEM and statistically significant differences between vaccine treatment groups (P value <0.05) are marked with *:*p*<0.05.

To further evaluate whether antibodies obtained from seronegative pigs (Study I) could inhibit the attachment of the virus to the chicken red blood cells (RBCs), an HAI assay against the challenged virus was carried out. Albeit only detecting HAI activity at 7 dpi, results displayed in Fig 5 suggest that all swine from the vaccinated group had significantly higher detectable inhibition of the hemagglutination (HI) than the non-vaccinated pigs (*p*<0.05). None of the pigs vaccinated with pCMV-CTLA4-Ig (empty plasmid) could inhibit the hemagglutination of SwH3N2 (S1 Table).

### Virus detection in BALFs

**Fig 6:**
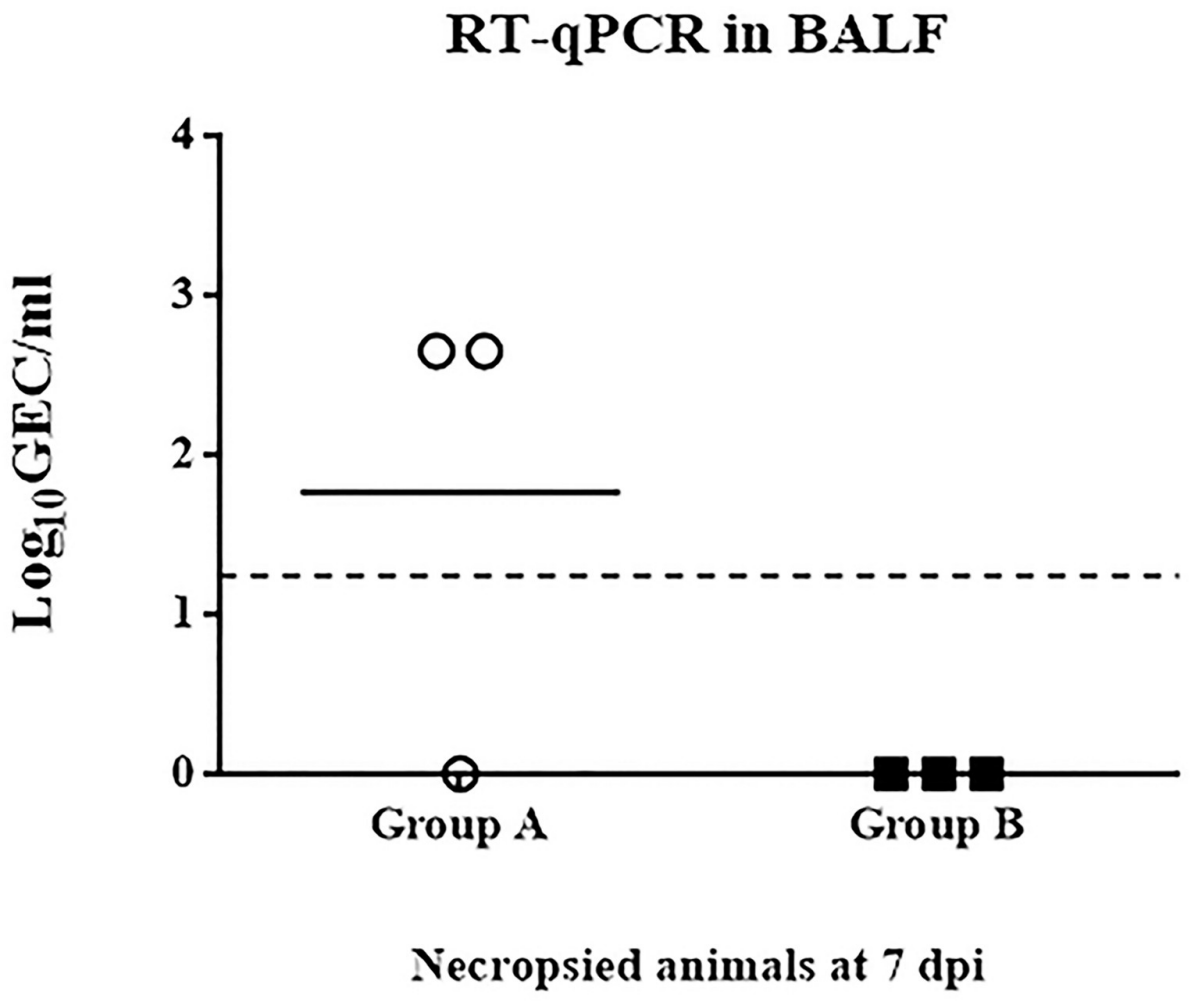
Influenza viral RNA detection in BALFs performed by RT-qPCR. GEC per mL values obtained in BALFs samples, obtained from MDA positive animals (Study 2), from unvaccinated (Group A) or vaccinated (Group B) pigs at 7 dpi, corresponding to necropsy day. White circles designate group A (unvaccinated group), and black squares designate group B (pCMV-CTLA4-Ig-NG34 vaccinated group). Dashed lines indicate the detection limit of the assay: 1.24 log_10_GEC/mL.

BALFs collected in the second study were also used to assess whether at 7 dpi and 14 dpi SIV RNA could be detected in the respiratory tract of the lungs. While no viral RNA could be detected at 14 dpi in any of the vaccinated pigs, differences between groups were evident at 7 dpi. Influenza virus RNA could not be detected in any of the BALFs collected from the vaccinated pigs. In contrast, BALFs from two out of the three non-vaccinated pigs were positive for SIV RNA (Fig 6), demonstrating a clearance of viral RNA in the respiratory airways from the pCMV-CTLA4-Ig-NG34 vaccinated pigs.

Data gathered from the complementary study showed that none of the three pigs vaccinated with pCMV-CTLA4-Ig (empty plasmid) cleared the infection in the lung (S1 Table).

## Discussion

Conserved peptides are highly desirable vaccine antigens/candidates for various reasons [36], particularly with regard to safety and ease of production. NG34, the peptide antigen used in this study, is relatively conserved and, as we reported previously [25], it consists of both a B and T cell epitope. For this dual role of the peptide fomenting both humoral and cellular responses in conventional pigs and the demonstrated *in vitro* cross-protective immune reaction [25], we are seeking new strategies to present the antigen in a formulation that can potentiate the immune response and confer protection against influenza virus infection. For this purpose, NF-34 was modified to NG34. Immunization of mice with this modified HA-peptide (NG34) elicited sustained antibodies with strong neutralizing capacity [37]. Moreover, by cloning NG34 into the pCMV-CTLA4-Ig plasmid, our intention was to target the antigen presenting cells with the objective to induce an enhanced immune response. CTLA4 was chosen due to its described adjuvant like role at low doses as it delivers fused antigens to antigen-presenting-cells (APCs) [38]. Data reported in mice [38] indicated that targeting antigens to antigen presenting cells (APCs) by means of CTLA4 increased both the humoral and the cellular responses. The role of CTLA4-Ig as an adjuvant has also been reported in other studies including a model of asthma [39]. Although it has been described that IgG2a production is predominant after DNA immunization [40], mice immunized with DNA-CTLA4-Ig generated enhanced levels of distinct IgG subclasses (IgG1, IgG2a, IgG2b), with a predominance of the IgG1 subtype [38]. This suggests that CTLA4 might have caused a non-specific change in the immune response, possibly by a direct stimulation of APCs.

An intradermal delivery approach was chosen for vaccine prototype delivery mainly owing to a large number of studies [41–43] warranting higher antibody titers by this method in comparison to gene injection into skeletal muscles. Furthermore, using this approach we facilitated DNA uptake by skin-associated-lymphoid tissue that may play a role in inducing cytotoxic T cells against viruses or intracellular pathogens [42]. We used the DNA vaccine delivery approach first described by [44] which was previously applied also using another influenza DNA vaccine in pigs with challenge [45]. Optimal influenza DNA plasmid doses (moles) were identified and suggested using this delivery method in pigs [27]. Using the same delivery method in pigs but another multivalent influenza DNA vaccine we also were able to break MDA and protect pigs from influenza challenge. Since the DNA vaccine and the challenge strain (H1N1psm2009) were different, we cannot compare. However, the influenza DNA studies both suggest that naked DNA vaccine seems all very powerful in protecting pigs from heterologous influenza strains of both H1N1 and H3N2. Similar results have been consistently reported by others using different influenza DNA and delivery methods [46,47].

Introduction of the NG34 peptide sequence into plasmid together with CTLA4 further improved the immunogenicity and protective potential of the peptide-based vaccine previously reported by our group [25]. Moreover, seeing the complementary study data using pCMV-CTA4-Ig it is evident that the combination of CTLA4 with NG34 is fundamental. An additional DNA adjuvant effect may be obtained using the Diluvac diluent containing Tocopherol [48]. Vaccinated animals completely eliminated virus from the lung within 7 days after challenge as demonstrated from BALFs samples collected from seropositive pigs. Additionally, in seronegative vaccinated animals, viral shedding was also reduced to basic levels within 5 to 7 days after infection, suggesting that transmission of the virus could greatly be reduced with the vaccine approach used. Interestingly, vaccinated MDA positive could also reduce virus replication and shedding, suggesting that pCMV-CTLA4-Ig-NG34 vaccine could overcome a possible inhibition/delay in inducing an active antibody and/or cellular immune response [19,27]. The virus clearing effects could apparently be linked to CTLA4-Ig vaccination that is involved in IgG1 activity promoting Th2 response, in a possible transportation of the antigens in lymphoid organs [49] and in an increase of the B cell and T cell response [38]. In addition, elevated levels of anti-HA specific antibodies at 35 PVD and 7days after a H3N2 inoculation in the vaccinated pigs might have played a role in the elimination of the heterologous challenged virus. Additionally, these antibodies potently inhibited the hemagglutination activity of the challenged virus at 7 dpi. Moreover and as reported in [25] vaccination induced and maintained antibody cross-reactive response against H3N2 and H1N1 subtype. Furthermore, a tendency of a higher IgG titer in BALFs against H3N2 and H1N1 subtypes was observed in the vaccinated MDA animals compared to the non-vaccinated ones.

Clinical signs and lung lesions were similar between groups. However, in the first study, more animals from the vaccinated groups had fever than the animals from the unvaccinated group. Likewise, there was one seropositive pig from the unvaccinated group (2^nd^ study, animal 11) that cleared the virus at 7 dpi, at least based on viral RNA presence in BALFs samples. This animal coincided to have had the highest MDA levels of the group at the onset of the experiment. Besides, due to the small number of samples studied for SIV RNA and antibody titers in BALFs, no statistical analyses were performed.

In summary, intradermal application of pCMV-CTLA4-Ig-NG34 DNA vaccine might represent a potential alternative to combat SIVs and could overcome MDA-associated blockage of the virus secretion. We anticipate that reducing/blocking/eliminating the influenza virus shedding after infection is crucial for concomitant transmission to indirect naïve contact pigs. Nonetheless, more studies are indispensable (with larger groups) and might be mainly addressed to examine whether the presented formulation is also capable of promoting a solid response against other widely circulating swine influenza subtypes.

## Supporting information

S1 Table Summary of the complementary study results using pCMV-CTLA4-Ig plasmid. (PDF)

S2 Table Mean and mean of the standard deviation of the GEC per mL of the nasal swabs samples collected from the 1^st^study at 0, 3, 5 and 7. (PDF)

S3 Table Mean and mean of the standard deviation of the GEC per mL from subtypic RT-qPCR of the nasal swabs samples collected from the 1^st^study at 0, 3, 5 and 7. (PDF)

S4 Table Mean and mean of the standard deviation of the GEC per mL of the nasal swabs samples collected from the 2^nd^study at 0, 1, 2, 4 and 7. (PDF)

S5 Table Mean and mean of the standard deviation of the GEC per mL from subtypic RT-qPCR of the nasal swabs samples collected from the 2^nd^study at 0, 1, 2, 4 and 7. (PDF)

S6 Table Mean and standard deviations of OD 450 nm values obtained against HA of A/California/04/09(H1N1)pdm09 from sera samples for each triplicate at 0, 20PVD, 35PVD and 7 dpi. (PDF)

S7 Table Mean and standard deviation of OD 450 nm values obtained against HA from A/Aichi/2/1968(H3N2) from sera samples for each triplicate at 0, 20PVD, 35PVD and 7 dpi. (PDF)

S8 Table Mean and standard deviation of OD 450 nm values obtained against HA from A/California/04/09(H1N1)pdm09 from BALFs samples for each triplicate at 7 and 14 dpi. (PDF)

S9 Table Mean and standard deviation of OD 450 nm values obtained against HA from A/Aichi/2/1968(H3N2) from BALFs samples for each triplicate at 7 and 14 dpi. (PDF)

S10 Table Individual animal mean HI titer obtained against virus A/swine/Spain/003/2010 H3N2 IV from sera samples for each duplicate at 7 dpi. (PDF)

S11 Table GEC per mL of the BALFs samples collected from the 2^nd^ study at 7 dpi. (PDF)
