# Supplementary material for: Conserved HA-peptide NG34 formulated in pCMV-CTLA4-Ig reduces viral shedding in pigs after a heterosubtypic influenza virus SwH3N2 challenge
Source: PLoS One. 2019 Mar 1;14(3):e0212431. doi: 10.1371/journal.pone.0212431 (PMC6396909; doi:10.1371/journal.pone.0212431)
Supplement: S1 Table — (PDF) [file pone.0212431.s001.pdf]

S1 Table. Summary of results obtained in a preliminary trial performed in pigs vaccinated with pCMV-CTLA4-Ig (empty vector) and challenged with H3N2 SIV.

Assay		
	Mean	Mean SD
Virus quantification by RT-qPCR in NS	2,80[a]	0,526
Virus quantification by RT-qPCR in BALFs	4,01[b]	0,535
ELISA for H3-antibody detection	0,35[c]	0,079
HI test	0,00[d]	0,00

Abbreviations: BALFs, bronchoalveolar fluids; dpi, days post-inoculation; GEC, genomic equivalent copies; HA, hemagglutinin; HI,hemagglutination inhibition; IV, influenza virus; NS, nasal swabs; OD, optical density; PVD, post-vaccination day; SIV, swine influenza virus.

[a]Mean Log_{10} GEC/mL of NS collected from animals immunized with pCMV-CTLA4-Ig at 7 dpi with 10^6 $TCID_{50}$/mL H3N2 SIV.

[b]Mean Log_{10} GEC/mL of the BALFs from animals immunized with pCMV-CTLA4-Ig at 7 dpi with 10^6 $TCID_{50}$/mL H3N2 SIV.

[c]Mean values against HA (A/Aichi/2/1968(H3N2)) from sera samples at 35 PVD obtained by ELISAs.

[d]Mean HI titers against A/swine/Spain/003/2010 H3N2 IV from sera samples at 7 dpi with $10^6$$TCID_{50}$/mL H3N2 SIV.
