# Supplementary material for: Conserved HA-peptide NG34 formulated in pCMV-CTLA4-Ig reduces viral shedding in pigs after a heterosubtypic influenza virus SwH3N2 challenge
Source: PLoS One. 2019 Mar 1;14(3):e0212431. doi: 10.1371/journal.pone.0212431 (PMC6396909; doi:10.1371/journal.pone.0212431)
Supplement: S3 Table — (PDF) [file pone.0212431.s003.pdf]

	Viral shedding in nasal swabs (1st study)			
	Group A- Unvaccinated group		Group B- pCMV-CTLA4-Ig-NG34	
**Time-point**	**Mean Log_{10}GEC/mL**	**Mean SD**	**Mean Log_{10}GEC/mL**	**Mean SD**
0	Negative	Negative	Negative	Negative
3	3,91	1,49	4,29	1,56
5	2,73	1,72	1,83	0,99
7	Negative	Negative	Negative	Negative

S3 Table. Mean and mean of the standard deviation of the genome equivalent copies (GEC) per mL from the subtypic RT-qPCR of the nasal swabs samples collected from the 1st study at 0, 3, 5 and 7.
