# Supplementary material for: Conserved HA-peptide NG34 formulated in pCMV-CTLA4-Ig reduces viral shedding in pigs after a heterosubtypic influenza virus SwH3N2 challenge
Source: PLoS One. 2019 Mar 1;14(3):e0212431. doi: 10.1371/journal.pone.0212431 (PMC6396909; doi:10.1371/journal.pone.0212431)
Supplement: S4 Table — (PDF) [file pone.0212431.s004.pdf]

	Viral shedding in nasal swabs (2nd study)			
	Group A- Unvaccinated group		Group B- pCMV-CTLA4-Ig-NG34	
Time-point	Mean Log_{10}GEC/mL	Mean SD	Mean Log_{10}GEC/mL	Mean SD
0	Negative	Negative	Negative	Negative
1	1,24	0,03	1,28	0,11
2	1,79	0,84	2,57	1,38
4	3,89	1,09	3,14	1,17
7	2,46	0,29	1,77	0,51

S4 Table. Mean and mean of the standard deviation of the genome equivalent copies (GEC) per mL of the nasal swabs samples collected from the 2nd study at 0, 1, 2, 4 and 7.
