# Supplementary material for: Conserved HA-peptide NG34 formulated in pCMV-CTLA4-Ig reduces viral shedding in pigs after a heterosubtypic influenza virus SwH3N2 challenge
Source: PLoS One. 2019 Mar 1;14(3):e0212431. doi: 10.1371/journal.pone.0212431 (PMC6396909; doi:10.1371/journal.pone.0212431)
Supplement: S5 Table — (PDF) [file pone.0212431.s005.pdf]

	Viral shedding in nasal swabs (2nd study)			
	**Group A- Unvaccinated group**		**Group B- pCMV-CTLA4-Ig-NG34**	
**Time-point**	**Mean Log_{10}GEC/mL**	**Mean SD**	**Mean Log_{10}GEC/mL**	**Mean SD**
0	Negative	Negative	Negative	Negative
1	1,84	1,48	2,29	1,11
2	4,27	2,08	5,15	1,08
4	5,63	1,54	5,30	0,85
7	2,78	1,19	2,05	0,68

S5 Table. Mean and mean of the standard deviation of the genome equivalent copies (GEC) per mL from subtypic RT-qPCR of the nasal swabs samples collected from the 2nd study at 0, 1, 2, 4 and 7.
