# Supplementary material for: Conserved HA-peptide NG34 formulated in pCMV-CTLA4-Ig reduces viral shedding in pigs after a heterosubtypic influenza virus SwH3N2 challenge
Source: PLoS One. 2019 Mar 1;14(3):e0212431. doi: 10.1371/journal.pone.0212431 (PMC6396909; doi:10.1371/journal.pone.0212431)
Supplement: S6 Table — (PDF) [file pone.0212431.s006.pdf]

	Anti-rH1pdm09 OD 450nm values in sera (1st study)			
	Group A- Unvaccinated group		Group B- pCMV-CTLA4-Ig-NG34	
**Time-point**	**Mean**	**SD**	**Mean**	**SD**
0	0,182	0,076	0,313	0,135
20 PVD	0,224	0,032	0,348	0,087
35 PVD	0,172	0,011	1,436	0,058
7 DPI	0,191	0,031	1,378	0,021

S6 Table. Mean and standard deviations of OD 450 nm values obtained against HA of A/California/04/09(H1N1)pdm09 from sera samples for each triplicate at 0, 20 PVD, 35 PVD and 7 dpi.
