# Supplementary material for: Conserved HA-peptide NG34 formulated in pCMV-CTLA4-Ig reduces viral shedding in pigs after a heterosubtypic influenza virus SwH3N2 challenge
Source: PLoS One. 2019 Mar 1;14(3):e0212431. doi: 10.1371/journal.pone.0212431 (PMC6396909; doi:10.1371/journal.pone.0212431)
Supplement: S7 Table — (PDF) [file pone.0212431.s007.pdf]

	Anti-rH3 1968 OD 450nm values in sera (1st study)			
	Group A- Unvaccinated group		Group B- pCMV-CTLA4-Ig-NG34	
**Time-point**	**Mean**	**SD**	**Mean**	**SD**
0	0,264	0,008	0,293	0,017
20 PVD	0,359	0,022	0,478	0,013
35 PVD	0,259	0,168	0,890	0,044
7 DPI	0,340	0,013	2,614	0,147

S7 Table. Mean and standard deviation of OD 450 nm values obtained against HA of A/Aichi/2/1968(H3N2) from sera samples for each triplicate at 0, 20 PVD, 35 PVD and 7 dpi.
