# Supplementary material for: Conserved HA-peptide NG34 formulated in pCMV-CTLA4-Ig reduces viral shedding in pigs after a heterosubtypic influenza virus SwH3N2 challenge
Source: PLoS One. 2019 Mar 1;14(3):e0212431. doi: 10.1371/journal.pone.0212431 (PMC6396909; doi:10.1371/journal.pone.0212431)
Supplement: S8 Table — (PDF) [file pone.0212431.s008.pdf]

| | Anti-rH1pdm09 OD 450nm values in BALF (2nd study) | | | |
|---|---|---|---|---|
| | Group A- Unvaccinated group | | Group B- pCMV-CTLA4-Ig-NG34 | |
| **Time-point** | **Mean** | **SD** | **Mean** | **SD** |
| 7 DPI | 0,390 | 0,113 | 0,447 | 0,005 |
| 14 DPI | 0,446 | 0,421 | 0,668 | 0,133 |

**S8 Table. Mean and standard deviation of OD 450 nm values obtained against HA of A/California/04/09(H1N1)pdm09 from BALF samples for each triplicate at 7 and 14 dpi.**
