# Supplementary material for: Conserved HA-peptide NG34 formulated in pCMV-CTLA4-Ig reduces viral shedding in pigs after a heterosubtypic influenza virus SwH3N2 challenge
Source: PLoS One. 2019 Mar 1;14(3):e0212431. doi: 10.1371/journal.pone.0212431 (PMC6396909; doi:10.1371/journal.pone.0212431)
Supplement: S9 Table — (PDF) [file pone.0212431.s009.pdf]

	Anti-rH3 1968OD 450nm values in BALF (2nd study)			
	**Group A- Unvaccinated group**		**Group B- pCMV-CTLA4-Ig-NG34**	
**Time-point**	**Mean**	**SD**	**Mean**	**SD**
7 DPI	0,408	0,049	0,628	0,057
14 DPI	0,895	0,234	1,424	1,007

S9 Table. Mean and standard deviation of OD 450 nm values obtained against HA of A/Aichi/2/1968(H3N2) from BALF samples for each triplicate at 7 and 14 dpi.
