# Supplementary material for: Conserved HA-peptide NG34 formulated in pCMV-CTLA4-Ig reduces viral shedding in pigs after a heterosubtypic influenza virus SwH3N2 challenge
Source: PLoS One. 2019 Mar 1;14(3):e0212431. doi: 10.1371/journal.pone.0212431 (PMC6396909; doi:10.1371/journal.pone.0212431)
Supplement: S10 Table — (PDF) [file pone.0212431.s010.pdf]

Against H3N2 2010 HI titer in sera (1st study)			
**Animal**	**Group A- Unvaccinated group**	**Animal**	**Group B- pCMV-CTLA4-Ig-NG34**
1	20	6	160
2	20	7	320
3	*	8	40
4	30	9	320
5	160	10	320

*Animal 3 died at 2 days post-inoculation

S10 Table. Individual animal mean HI titer obtained against virus A/swine/Spain/003/2010 H3N2 IV from sera samples for each duplicate at 7 dpi.
