# Supplementary material for: Conserved HA-peptide NG34 formulated in pCMV-CTLA4-Ig reduces viral shedding in pigs after a heterosubtypic influenza virus SwH3N2 challenge
Source: PLoS One. 2019 Mar 1;14(3):e0212431. doi: 10.1371/journal.pone.0212431 (PMC6396909; doi:10.1371/journal.pone.0212431)
Supplement: S11 Table — (PDF) [file pone.0212431.s011.pdf]

Viral shedding in BALF (2nd study)			
**Group A- Unvaccinated group**		**Group B- pCMV-CTLA4-Ig-NG34**	
**Animal**	**$Log_{10}GEC/mL$**	**Animal**	**$Log_{10}GEC/mL$**
1	2,65	7	Negative
2	2,65	9	Negative
5	Negative	12	Negative

S11 Table. Genome equivalent copies (GEC) per mL of the BALF samples collected from the 2nd study at 7 dpi.
